# *SLC26A4* mutation in Pendred syndrome with hypokalemia: A case report

**DOI:** 10.1097/MD.0000000000030253

**Published:** 2022-09-02

**Authors:** Ya-Ting Lu, Lin Wang, Le-Le Hou, Ping-Ping Zheng, Qian Xu, Da-Tong Deng

**Affiliations:** a Department of Endocrinology, The First Affiliated Hospital of Anhui Medical University, Hefei, Anhui Province, China; b Department of Endocrinology, Zhongda Hospital, Medical School, Southeast University, Nanjing, Jiangsu Province, China.

**Keywords:** case report, gene mutation, hypokalemia, Pendred syndrome, pendrin

## Abstract

Pendred syndrome is an autosomal recessive disorder characterized by sensorineural hearing loss, inner ear malformations, goiter, and abnormal organification of iodide. It is caused by mutations in *SLC26A4* gene, which encodes pendrin (a transporter of chloride, bicarbonate, and iodide). Pendred syndrome is a common cause of syndromic deafness, but the metabolic abnormalities it causes are often overlooked. Here, we report the case of a patient diagnosed with Pendred syndrome with hypokalemia.

**Patient concerns:**

A 53-year-old deaf-mute woman was hospitalized due to severe limb asthenia. The emergency examination showed that her blood potassium level was 1.8 mmol/L.

**Diagnoses:**

Through the genetic test, we found a mutation of SLC26A4 gene in NM_000441: c.2027T>A, p.L676Q, as well as the *SLC26A4* exon 5-6 deletion. These genetic variations pointed to Pendred syndrome (an autosomal recessive disorder that mainly affects the inner ear, thyroid, and kidney) which is a common cause of syndromic deafness.

**Interventions:**

The patient was treated with potassium supplements and screened for the cause of hypokalemia.

**Outcomes:**

The patient was discharged after her potassium levels rose to the normal range.

**Lessons:**

Patients with Pendred syndrome may also have certain metabolic abnormalities; thus, more attention should be paid to them during clinical diagnosis.

## 1. Introduction

Pendred syndrome is an autosomal recessive disorder characterized by sensorineural hearing loss, and inner ear malformations such as an enlarged vestibular aqueduct, goiter, and abnormal organification of iodide.^[1]^ It is caused by mutations in *SLC26A4* gene, which encodes pendrin (a transporter of chloride, bicarbonate and iodide).^[2]^ Pendrin is not only expressed in the inner ear and thyroid, but also plays an important role in mediating the secretion of bicarbonate into the kidney.^[3,4]^ Therefore, in the absence of pendrin, bicarbonate retention and metabolic alkalosis may occur. However, the diagnostic role of *SLC26A4* (pendrin) mutation in Pendred syndrome with hypokalemia remains to be clarified.

## 2. Case presentation

On February 6, a 53-year-old woman was admitted to the hospital. The patient suffered from deafness before learning speech or language and has a history of goiter for >20 years. In September 2017, she underwent thyroidectomy. After the operation, she was treated with 50 mg QD of levothyroxine sodium tablet, and her thyroid function was reexamined. Later, she gradually became mental apathy and limb weakness. In the last 3 days, these symptoms were aggravated and finally she was unable to walk. Then, she was referred to the emergency department of our hospital. The results of laboratory testing showed that her blood potassium level was 1.8 mmol/L, and blood calcium level was 1.45 mmol/L. After treatment with intravenous and oral potassium supplements, a normal blood potassium level of 3.97 mmol/L was achieved, and the symptoms were relieved. To further clarify the cause of hypokalemia, the patient was transferred to the Department of Endocrinology. The family members reported that they had no symptoms related to digestive tract, such as sweating, hunger and satiety, nausea, vomiting, abdominal pain and diarrhea, and no significant change in recent body weight. Her family history is as follows: her parents are both healthy, without any blood relatives besides their children; she has an elder sister and a younger brother; at present, the former is also deaf-mute, but the latter is healthy; and a history of hypokalemia is denied for her brother.

Physical examination showed that her blood pressure was 128/87 mm Hg; her height, weight, and BMI were 158 cm, 68 kg, and 27.2 kg/m^2^, respectively; her heart rate was 100 beats/min; her thyroid gland did not enlarge; limb muscle strength was level 5-; and muscle tension was normal. After hospitalization, the levels of thyroid hormone, parathyroid hormone (10.50 pg/mL), antinuclear antibody, and antineutrophil cytoplasmic antibody were all normal. The results of 24-hour urine electrolyte showed that the urinary levels of potassium, sodium, chlorine, calcium, phosphorus, magnesium were 40.2 mmol/L (normal range = 25–100 mmol/L), 274 mmol/L (↑; normal range = 130–260 mmol/L), 310 mmol/L (↑; normal range = 170–250 mmol/L), 2.10 mmol/L (↓; normal range = 2.5–7.5 mmol/L), 10.50 mmol/L (↓; normal range = 22–48 mmol/L), and 2.04 mmol/L (↓; normal range = 2.1–8.2 mmol/L), respectively, during the 24-hour collection period. The results of erect position and clinostatism tests are shown in Table 1. The ECG showed that Q-T interval was prolonged, and adrenal computed tomography enhanced scan revealed no significant abnormality.

**Table 1:** Results of the patient’s erect position and clinostatism tests.

	Aldosterone (pmol/L)	Angiotensin-I (pg/mL)	Angiotensin-II (pg/mL)	Renin (ng/mL/h)	Cortisol (nmol/L)
Reference value	Decubitus: 124–483	0.05–0.79	28.2–52.2	0.56–2.8	138.0–690.0
	Erect position: 270–759				
Decubitus	410.20	1.40	70.30	0.90	170.51
Erect position (10:00 am)	598.20	2.90	97.60	1.00	
Erect position (12:00 am)	620.70	2.80	90.30	0.90	457.82
ARR	21.60				

**Figure 1.**
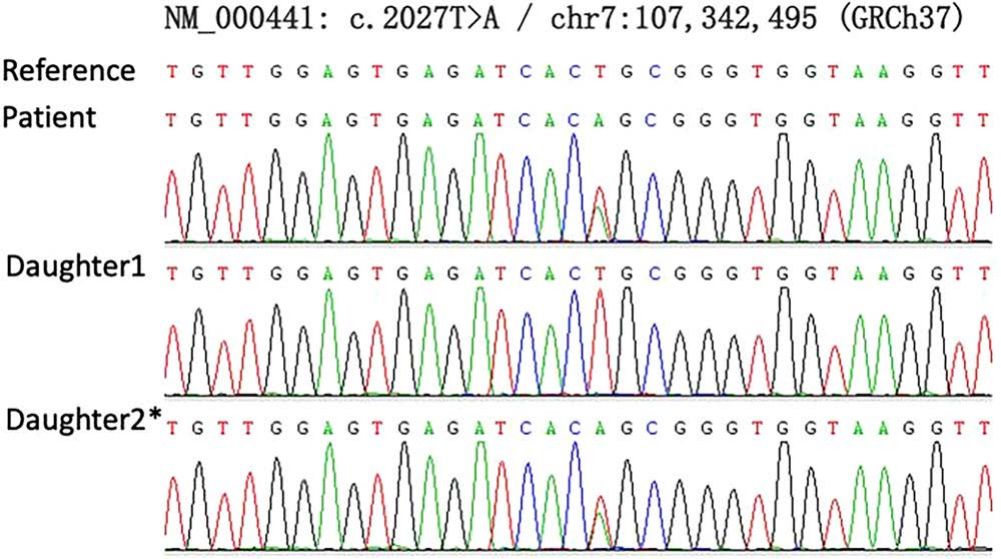
Results of *SLC26A4* mutation analysis (* Carrier). Sanger sequencing confirmed c.2027T>A in the patient and her second daughter.

**Figure 2.**
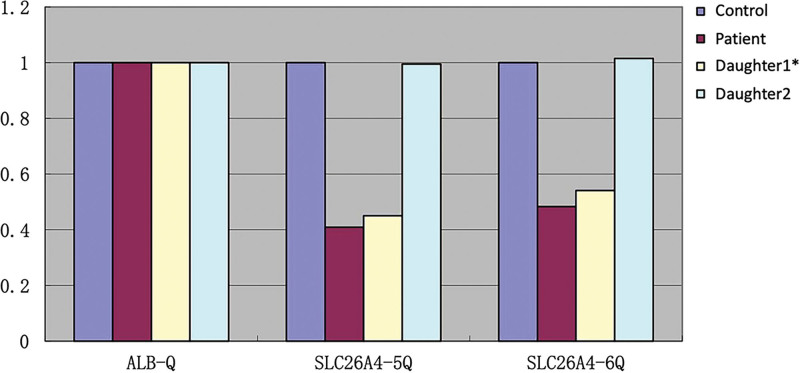
Results of quantitative real-time PCR assays (* Carrier). The contrast ratio of *SLC26A4* gene copy number in the propositus second daughter and normal control was approximately 1, while the contrast ratios in the propositus, the propositus eldest daughter, and normal control were about 0.5, indicating that the hybrid *SLC26A4* exon 5-6 is an absence in the propositus and the propositus eldest daughter. PCR = polymerase chain reaction.

**Figure 3.**
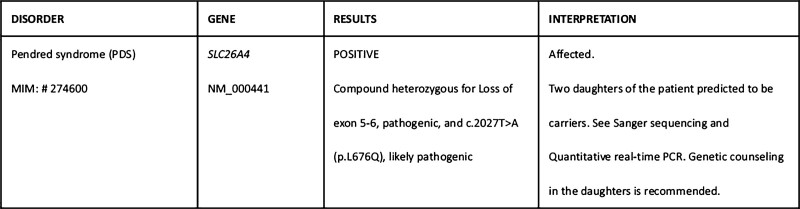
Results of the patient’s genetic test.

According to the patient’s medical history and examination results, the patient had a slight loss of appetite, no vomiting, diarrhea, excessive sweating, and no previous paroxysmal skeletal muscle flaccid paralysis. Her 24-hour urine potassium and adrenal computed tomography enhanced scan were normal, so common causes of hypokalemia were excluded. Considering the patient’s congenital deafness and goiter, we did genetic testing on the patient and her daughters and identified compound heterozygous variations, exon 5-6 deletion, and NM_000441: c.2027 (exon 17)T>A, p.L676Q, in the *SLC26A4* gene by whole exome sequencing. Then, we confirmed that both the proband and the second daughter were heterozygous, while the eldest daughter was wild-type by Sanger sequencing (Fig. 1). The laboratory conducted the same group of fluorescence quantitative polymerase chain reaction assays for the healthy control samples as well as the propositus and daughter samples, and detected the copy number of the exon 5-6 of target gene *SLC26A4* with *ALB* gene as the internal reference gene. The results demonstrated that the contrast ratio of *SLC26A4* gene copy number in the propositus second daughter and normal control was approximately 1 (Fig. 2), suggesting that *SLC26A4* exon 5-6 copy number is normal for the propositus second daughter. On the contrary, the contrast ratios of *SLC26A4* gene copy number in the propositus, the propositus eldest daughter, and normal control were about 0.5 (Fig. 2), indicating that the hybrid *SLC26A4* exon 5-6 is an absence in the propositus and the propositus eldest daughter. In summary, this patient was considered to be diagnosed with Pendred syndrome with hypokalemia and both the daughters were carriers (Fig. 3). We asked the patient’s 2 daughters about their histories, and neither of them had any hearing loss, goiter, or limbs muscle weakness. At the same time, the arterial blood gas analysis and electrolytes of these 3 people were examined, as shown in Table 2. The results showed that the second daughter of the patient had normal blood potassium and sodium, while the eldest daughter had decreased potassium and sodium. Neither of them had bicarbonate retention or metabolic alkalosis.

**Table 2:** Results of arterial blood gas and electrolyte measurement.

	pH	PCO_2_ (mm Hg)	HCO_3_− (mmol/L)	BE	K^+^ (mmol/L)	Na^+^ (mmol/L)	Cl− (mmol/L)
Proband	7.510↑	40.5	32.6↑	9.3↑	2.86↓	140.8	93.5↓
Eldest daughter	7.442	27.7↓	19.0↓	-5.3↓	3.48↓	136.2↓	102.8
Second daughter	7.416	33.7↓	21.8↓	-3	3.84	142.7	101.2

The patient was discharged after her potassium levels rose to the normal range and the cause of hypokalemia was clarified. We told the patient to monitor blood potassium regularly after discharge.

## 3. Discussion

Hypokalemia is a common electrolyte disorder in clinic. There are a number of factors that can trigger such metabolic imbalance. The most common factor is the extrarenal loss of potassium, which is partly caused by a decrease in potassium intake or cell migration. According to the clinical differentiation of hypokalemia, we should consider the following causes: gastrointestinal potassium loss; use of diuretics; hypomagnesemia; Bartter syndrome; and Gitelman syndrome. Thus, in the near future, it is necessary to ask the family members whether the patients have significant changes in appetite and body weight. Potassium is commonly found in all kinds of food. It is not easy to induce hypokalemia due to insufficient potassium intake in a short period of time. Typically, hypokalemia can only occur when the daily intake of potassium chloride is <3 g and it lasts for >2 weeks. In this study, the patient did not have obvious symptoms of gastrointestinal potassium loss, such as diarrhea, and denied taking diuretics. Therefore, hypokalemia caused by insufficient potassium intake, gastrointestinal potassium excretion, and other drug factors is not considered. Since the patient was deaf at her early ages, and both Bartter and Gitelman syndromes were rare autosomal recessive disorders, the patient and her 2 daughters were genetically tested. We found a mutation of *SLC26A4* gene in NM_000441: c.2027T>A, p.L676Q, as well as the *SLC26A4* exon 5-6 deletion. These genetic variations pointed to Pendred syndrome.

Pendred syndrome is thought to account for about 10% of hereditary hearing loss and is one of the most common forms of syndromic deafness.^[5]^ In 1997, it was reported that the double allele mutation of *SLC26A4* gene (also known as Pendred syndrome gene) could explain the cause of this syndrome at the molecular level.^[6]^ The gene encodes pendrin, a multifunctional anion transporter. The mRNA expression of *SLC26A4* was found to be highly abundant in the kidney, especially the renal cortex.^[7]^ Both immunohistochemical and immunoblot studies indicated that pendrin was localized at the apical brush-border membrane in type B and non-A/non-B intercalated cells.^[3,8]^ More notably, pendrin could act as an apical exchanger of chloride with bicarbonate in type B intercalated cells.

Animal experiments^[9]^ showed that, in response to salt restriction, which led to an increase in aldosterone levels, the urinary volume and excretion of chloride were higher in Slc26a4−/− mice than in wild-type mice. Slc26a4−/− mice also had higher serum bicarbonate levels than wild-type mice, possibly due to the impaired ability to excrete hydroxide equivalents in response to salt restriction. Moreover, Slc26a4−/− mice were unable to retain sodium during dietary salt restriction because of a low abundance and diminished function of the sodium channel EnaC.^[10]^ Our patient had a higher level of aldosterone beyond the normal range, along with the low blood potassium and chlorine levels, and her blood sodium levels were within the normal range but relatively low. Her blood bicarbonate concentration and 24-hour urinary chloride excretion were both increased. These findings indicated that she had similar metabolic characteristics with the animal model. Besides, the patient and her eldest daughter harbored a heterozygous *SLC26A4* deletion, and the eldest daughter also exhibited decreased levels of potassium and sodium. Therefore, it can be speculated that the clinical manifestations of hypokalemia are associated with *SLC26A4* gene mutation.

Previous reports on patients with Pendred syndrome had mostly described deafness, goiter, and thyroid dysfunction, and few patients had been reported to develop any significant acid-base and electrolyte disturbances. Hypokalemia and metabolic alkalosis were reported in only 3 patients with Pendred syndrome between 2008 and 2011.^[11–13]^ But unlike the patient we reported, these patients had a history of alcohol abuse, frequent vomiting, or diuretic use, which induced hypokalemia. Our patient had no other causes of hypokalemia except the mild loss of appetite after thyroidectomy, so we considered her hypokalemia to be a manifestation of Pendred syndrome-related metabolic abnormalities. It can be seen that patients with Pendred syndrome may also have underlying metabolic abnormalities which are not common clinically.

## 4. Conclusion

In conclusion, Pendred syndrome patients with *SLC26A4* mutation may have certain metabolic abnormalities. Thus, close attention should be paid to them during clinical diagnosis.

## Acknowledgment

The authors would like to express their gratitude to EditSprings (https://www.editsprings.com/) for the expert linguistic services provided.

## Author contributions

Conceptualization: Da-Tong Deng, Ya-Ting Lu, Lin Wang, Le-Le Hou.

Data curation: Ya-Ting Lu, Lin Wang, Ping-Ping Zheng.

Investigation: Ya-Ting Lu, Lin Wang, Le-Le Hou, Qian Xu.

Resources and supervision: Da-Tong Deng.

Writing – original draft: Ya-Tign Lu, Lin Wang.

Writing – review & editing: Da-Tong Deng.
